# Mode of action of granulocyte-colony stimulating factor (G-CSF) as a novel therapy for stroke in a mouse model

**DOI:** 10.1186/s12929-019-0597-7

**Published:** 2020-01-06

**Authors:** Jigar Modi, Janet Menzie-Suderam, Hongyuan Xu, Paola Trujillo, Kristen Medley, Michael L. Marshall, Rui Tao, Howard Prentice, Jang-Yen Wu

**Affiliations:** 1 Department of Biomedical Sciences, Charles E. Schmidt College of Medicine, Florida Atlantic University, Boca Raton, FL 33431 USA; 2 Center of Complex Systems and Brain Sciences, Florida Atlantic University, Boca Raton, FL USA; 3 Program in Integrative Biology, Florida Atlantic University, Boca Raton, FL 33431 USA; 4 AEURA Trust, 2525 Arapahoe Ave E4-138, Boulder, CO 80302 USA

**Keywords:** Granulocyte-colony stimulating factor (G-CSF), Bilateral common carotid artery occlusion (BCAO) model of stroke, Endoplasmic reticulum (ER) stress, Autophagy, Mitochondrial markers, Apoptosis

## Abstract

**Background:**

The FDA approved drug granulocyte-colony stimulating factor (G-CSF) displays anti-apoptotic and immunomodulatory properties with neurogenesis and angiogenic functions. It is known to demonstrate neuroprotective mechanisms against ischemic global stroke. Autophagy is a method for the degradation of intracellular components and in particular, unrestrained autophagy may lead to uncontrolled digestion of affected neurons as well as neuronal death in cerebral ischemia. Mitochondrial dynamics is vital for the regulation of cell survival and death after cerebral ischemia and an early upstream event in neuronal death is mitochondrial fission. We examined the pro-survival mechanisms of G-CSF against apoptosis resulting from autophagy, mitochondrial stress and endoplasmic reticulum (ER) stress.

**Methods:**

Male Swiss Webster mice (20 weeks of age) were subjected to bilateral common carotid artery occlusion (BCAO) for 30 min. After occlusion, mice were injected with G-CSF (50 μg/kg) subcutaneously for 4 days. Behavioral analysis was carried out using the corner test and locomotor activity test before animals were sacrificed on day 4 or day 7. Key proteins in ER stress, autophagy and mitochondrial stress induced apoptosis were analyzed by immunoblotting.

**Results:**

G-CSF improved neurological deficits and improved behavioral performance on corner and locomotor test. G-CSF binds to G-CSF receptors and its activation leads to upregulation of Akt phosphorylation (P-Akt) which in turn decreases levels of the ER stress sensor, GRP 78 and expression of proteins involved in ER stress apoptosis pathway; ATF6, ATF4, eIF2α, XBP1, Caspase 12 and CHOP. G-CSF treatment significantly decreased Beclin-1, an autophagy marker, and decreased mitochondrial stress biomarkers DRP1 and P53. G-CSF also up-regulated the mitochondrial fusion protein, OPA1 and anti-apoptotic protein Bcl-2 while down-regulating the pro-apoptotic proteins Bax, Bak and PUMA.

**Conclusions:**

G-CSF is an endogenous ligand in the CNS that has a dual activity that is beneficial both in reducing acute neuronal degeneration and adding to long-term plasticity after cerebral ischemia. G-CSF treatment exerts neuroprotective effects on damaged neurons through the suppression of the ER stress and mitochondrial stress and maintains cellular homeostasis by decreasing pro-apoptotic proteins and increasing of anti-apoptotic proteins.

## Introduction

One of the leading causes of disability and death is stroke. More than 87% of strokes are ischemic and caused by obstruction of one or more cerebral arteries [1]. An inadequate supply of oxygen and glucose results in an ischemic cascade involving mitochondrial [2] and endoplasmic reticulum (ER) [3] dysfunction.

Mitochondrial dynamics is vital for the regulation of cell survival and death; importantly, mitochondrial fission is an early upstream event in neuronal death after cerebral ischemia [4]. While the fission process involves the constriction and cleavage of mitochondria, the fusion process generates elongated mitochondria. Mitochondrial fission is regulated by a mitochondrial-binding GTPase, Dynamin-related protein 1 (DRP1) [5, 6]. Global cerebral ischemia causes a transient rise in the phosphorylation of DRP1 without affecting total DRP1 protein expression [6]. DRP1 is vital for mitochondrial fission and cell fate. In mitochondrial fusion, both the inner and outer membranes are controlled by numerous GTPase proteins, including optic atrophy protein 1 (OPA1) [7].

Autophagy is a biological, ordered, and destructive mechanism of the cell that helps to abolish unwarranted or dysfunctional components [8]. In addition, the special role of autophagy is to offer nutrients that maintain metabolism in reaction to the cellular nourishing conditions. Autophagy is vital for the preservation of intracellular homeostasis, but importantly unrestrained autophagy may lead to uncontrolled destruction of affected neurons in cerebral ischemia [6, 9, 10]. However, according to other studies, the combination of cerebral ischemia and hypoxia appears to be a much more powerful provocation of the autophagic and lysososmal cell death pathway than focal ischemia alone [11].

The mitochondrion plays crucial role in the cell death machinery because of its relationship to large list of apoptosis-related proteins [12]. Accumulating evidence suggests that a group of proteins of the B-cell lymphoma (BCL-2) family are strongly engaged in the regulation of neuronal death in cerebral ischemic stroke [13–15]. The BCL-2 protein family is a key regulator of outer mitochondrial membrane permeability and plays vital roles in the intrinsic apoptotic pathway [16]. The BCL-2 family has been categorized into 2 groups: anti-apoptotic proteins, including Bcl-2, Bcl-xL, and Bcl-w, and pro-apoptotic proteins, such as Bax, Bak, Bim, Bid, Bad, Noxa, and p53-upregulated modulator of apoptosis (PUMA) [16–19]. The pro-apoptotic BH3-only BCL-2 subfamily is known to be upregulated after cerebral ischemia [2, 20].

In the ER, ischemia elicits accumulation of unfolded proteins, with the successive stimulation of the unfolded protein response (UPR). The UPR involves the detection of unfolded protein by the intraluminal ER chaperone; glucose regulated protein 78 (GRP 78), which then separates from the three distinct ER stress transmembrane sensors; double-stranded RNA-activated protein kinase-like ER kinase (PERK), activating transcription factor 6 (ATF6), and inositol-requiring kinase 1 (IRE1), promoting their release [21]. Downstream functions of the PERK, ATF6 and IRE1 pathways will upregulate UPR genes and ER-associated degradation (ERAD) genes that will reduce the unfolded proteins in the ER [22]. However, in sustained ER stress the proapoptotic transcription factor, C/EBP homologous protein (CHOP) [23] is activated resulting in apoptosis.

With only limited progress in development of stroke treatments, it is therefore essential to acquire valuable neuroprotective agents to effectively treat stroke. G-CSF controls the generation, proliferation, survival, and maturation of neutrophilic granulocytes and provokes their release from bone marrow to the peripheral blood [23, 24]. G-CSF is an FDA approved hematopoietic growth factor and is currently employed in clinical practice to cure neutropenia caused by chemotherapy [25]. G-CSF displays several important functions including anti-apoptotic activity, immunomodulatory action, stimulates neurogenesis, and angiogenic capabilities [23].

We have used a mechanism based therapeutic approach for stroke to first examine the connection of mitochondrial, autophagy and endoplasmic reticulum stress inhibition in the protective action of G-CSF and second to analyze relevant ER stress pathways in the bilateral common carotid Artery occlusion (BCAO) model of stroke.

## Materials and methods

### Animal preparation

All animal procedures were carried out in accordance with the guidelines for care and use of Animals and were approved by the institutional animal care and use committee (IACUC) of the Florida Atlantic University, Boca Raton. Male Swiss Webster mice (20 weeks of age) were obtained from Charles River laboratory. Mice were anesthetized with ketamine (40 mg/kg, i.p.) plus xylazine (2 mg/kg, i.p). For maintenance of anesthesia, isoflurane concentration was used to 0.5%. Mice were breathing spontaneously via breathing mask throughout the surgical procedure. A rectal temperature probe was inserted. During surgery, mice were resting on a thermostat-controlled heating pad, ensuring a constant core temperature of 37.0 ± 0.5 °C.

### Bilateral common carotid artery occlusion (BCAO)

Anesthesia was maintained as described above. The mouse was placed on its back. The animal’s tail and paws were fixed to the heating pad using adhesive tape. A sagittal ventral midline incision (~ 1 cm length) was performed. Both salivary glands were carefully separated and mobilized to visualize the underlying both Common Carotid Artery (CCA). Both CCA’s were carefully separated from the respective vagal nerves and accompanying veins without harming these structures. Manipulations of the vagal nerves might lead to transient or permanent dysfunction of the parasympathetic nerve system, which has the potential for the occurrence of significant cardiac arrhythmia or even irreversible cardiac arrest. Therefore, it is crucial to avoid any manipulations of the vagal nerves. Both common carotid arteries (CCAs) were isolated, freed of nerve fibers, and occluded using non-traumatic aneurysm clips. Complete interruption of blood flow was confirmed under an operating microscope. After 30 min of ischemia, the aneurysm clips were removed from both CCAs and the incision was sutured closed. Post-surgery analgesics were administered, and animals could wake up within 10 to 20 min after surgery [26]. All mice subjected to BCAO exhibited a significant reduction in regional cerebral blood flow (RCBF) to 51% pre-BCAO values and the RCBF retuned to 91% of the pre-BCAO level at the beginning of reperfusion as monitored with a Laser Doppler Flowmeter (LDF). Restoration of blood flow (reoxygenation) was also observed directly under the microscope. Sham-operated controls were subjected to the same surgical procedures except that CCAs were not occluded [26]. The body temperature was monitored and maintained at 37 °C ± 0.5 °C during surgery and during the immediate postoperative period until the animals recovered fully from anesthesia.

### Corner tests

The corner test, which determines an animal’s asymmetric direction of turning when encountering a corner, is used as an indicator of brain injury. We used an experimental corner setup composed of two boards (with dimensions of 30 × 20 × 1 cm3) arranged to form a 30° corner; a small opening was left along the joint between the two boards. The mouse was placed 12 cm from the corner and allowed to walk into the corner, so that the vibrissae on both sides of the animal’s face contacted the two boards simultaneously [26]. Before BCAO procedure, we conducted behavior tests (stratification) on all mice to screen for mice with no turning asymmetry (*n* ≥ 18). Each mouse took part in ten trials, after which we calculated the percentage of turns to each side, recording only those turns involving full rearing along one of the boards [26]. This stratification procedure excludes mice with 80–100% asymmetric turns (*n* = 4); we included mice that turned in either direction (*n* = 14) with a pretest score of 0.50 ± 0.08. Each mouse took part in ten trials for up to 4 days after BCAO.

### Locomotion (force-plate actometer) test

The force-plate actometer is an ensemble of mechanical, electronic, and computing elements that embody mathematical and physical principles to produce measurement of whole-organism behavioral attributes of relevance to basic neuroscience research. Methods of calibration and details of data acquisition have been described elsewhere [27, 28]. Briefly, the force-plate actometer purchased from BASi Corp (model FPA-I; West Lafayette, IN, USA) consists of a force-sensitive plate at a resolution of 200 Hz, a sound attenuation chamber, a computerized data acquisition board, and an analysis system software (FPA 1.10.01). A newly-developed force-plate actometer was utilized to measure locomotor activity. Animals were placed on the force plate actometer for one separate 60 min sessions. Locomotor activity of BCAO mice with and without G-CSF treatment were done after 4 day and 7 days. Between each test, the plate was thoroughly cleaned with paper towels followed by a deodorant treatment (70% ethanol, 1% acetic acid, and then water) to remove animal waste (i.e., feces, urine, saliva, and furs) and odor. Trace data of movements were automatically stored on the hard drive for off-line analysis. Changes in locomotion were revealed through power spectral analysis and expressed as arbitrary distance. The unit for changes in the power force was arbitrary.

### Mice groups and treatment schedules

Animals were randomly assigned for sham, control, and experimental groups, divide equally for day 4 (*N* = 15) and day 7 (N = 15) experiment. Thereafter, in experimental group (G-CSF treated group, *N* = 10), rhG-CSF (Filgrastim: Akron, FL, USA) (50 μg/kg in 0.3 mL Dextrose 5%) was injected subcutaneously 30 min after occlusion and continue daily until the animal sacrificed on Day 4 and Day 7. In control groups (vehicle-treated group, *N* = 10), vehicle (0.3 ml Dextrose 5%) was injected subcutaneously 30 min after occlusion, followed by daily administration of the same dose for an additional 3 days. Mice were received vehicle for 4 days before sacrifice. Sham-operated group (N = 10) received the same surgical procedure without occlusion of common carotid artery. After surgery, animals could recover from the anesthesia and given food and water ad libitum. The animals were daily examined for body temperature and weight, and those who had body temperature more than 39 °C after 24 h were excluded from the experiment [29].

### TTC staining

Animals were deeply anesthetized by isoflurane (Phoenix) and decapitated, and then brains were rapidly removed. Using an adult mouse brain matrix (Matrix, Zivic Instruments), brains were sectioned coronally into 2-mm coronal slices (2, 4, 6, 8, and 10 mm from the frontal pole) and incubated for 5 min in a 2% (w/v) solution of 2,3,5-triphenyltetrazolium chloride (TTC; JT Baker, India) at 37 °C for staining followed by collecting samples for western blot [30, 31].

### Sample collection for western blot analysis

Animals were deeply anesthetized by isoflurane (Phoenix) and decapitated, and then brains were rapidly removed. After sacrifice, while the brain was on ice [30], Brains were sectioned coronally into 2-mm coronal slices (2, 4, 6, 8, and 10 mm from the frontal pole) by using brain matrix [30, 32] and collected samples divided in to Frontal (0–4 mm section) Middle (4–8 mm section) and Hind brain (8–10 mm section) sample for western blot. The sample of Frontal brain includes Striatum + Cortex parts and sample of Middle brain includes Hippocampus + surrounding structures whereas Hind brain sample involves Cerebellum. WB was primarily carried out on frontal and Middle section of brain. Samples homogenized in Lysis buffer consisting of 50 mM Tris–HCl, 150 mM NaCl, 2 mM EDTA, pH 8.0, 1% Triton-X-100, 1:100 dilution of mammalian protease inhibitors (Sigma-Aldrich, MO, USA) and protease inhibitor [33, 34] for immunoblotting. Protein concentrations of each sample solution were determined with a Bradford protein assay, and samples were stored at − 80^0^ C until use. Protein samples were separated by 12% sodium dodecyl sulfate–polyacrylamide gel electrophoresis and transferred onto nitrocellulose membranes. Western blot was carried out as described previously [33] with the following primary antibodies overnight: Abcam: GRP 78, OPA 1,DRP-1, G-CSF, XBP1, ATF4, Caspase-12 PUMA and P-53 (1:500); Cell Signaling: GAPDH (1: 3000), Akt, phosphorylated Akt (P- Akt), Beclin 1, Bax, Bak, eIF2α (1:500), Invitrogen: P-DRP1(1:500); Santa Cruz: CHOP/GADD153, G-CSFR, Bcl-2 (1: 1000); Imgenex: ATF6 (1:500) [Additional file 1: Table S1]. The membranes were washed three times with Tris-buffered saline containing 0.1% Tween-20 (TBS-T) and incubated with secondary antibodies for 1 h at room temperature. Secondary antibodies used were goat IRDye 800-conjugated anti-rabbit (1:15,000) and IRDye 680 conjugated anti-mouse (1: 15,000) antibodies (LI-COR Biosciences, Lincoln, NE, USA). Fluorescent signals were detected with a LI-COR Odyssey Fc system and the images were quantified with the provided either Image Studio 2.0 software or image J software [35].

### Data and statistical analysis

All data were expressed as the mean ± SEM. A computer program (SPSS 15.0, Chicago, IL, USA and Prism Graph Pad 7) was used for statistical analysis. The statistical significance of the data was determined with t-test or one-way ANOVA combined with Dunnett post-hoc or Tukey test for comparison between groups. Differences of *P* < 0.05 were considered statistically significant. At least three independent replicates were performed for each experiment.

## Results

### Effect of G-CSF treatment on G-CSFR, G-CSF protein and phosphorylated Akt (P-Akt) in BCAO mouse stroke model on day 7

**Fig. 1:**
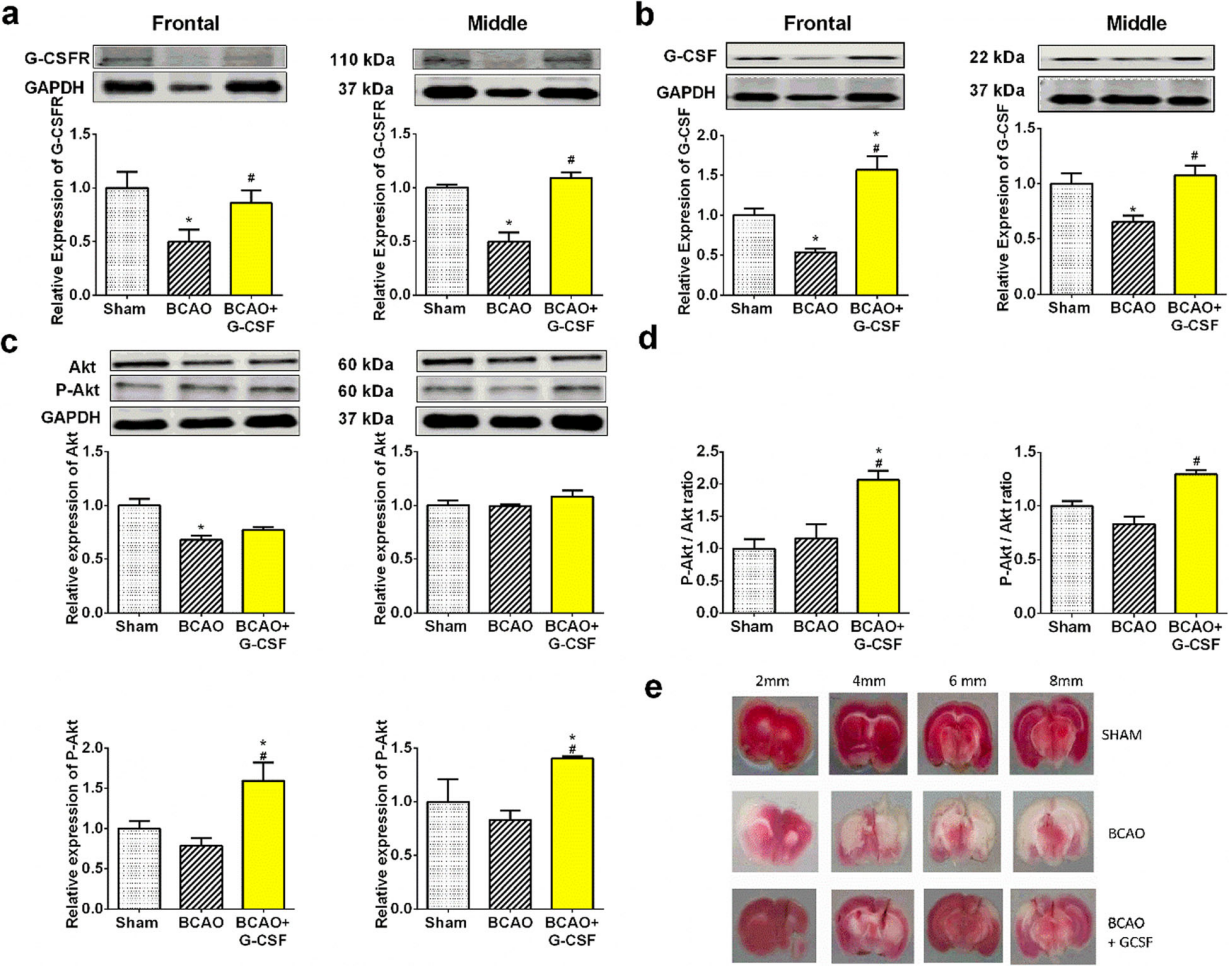
Effect of G-CSF on expression of G-CSFR, G-CSF and P-Akt proteins. **a** G-CSFR proteins levels in the frontal (F _(2, 8)_ = 7.519, *p* = 0.014) and middle (F _(2, 9)_ = 26.45, *p* = 0.0002) region of the brain. **b** G-CSF proteins levels in the frontal (F _(2, 20)_ = 18.58, *p* < 0.0001) and middle (F _(2, 14)_ = 6.319, *p* = 0.0111) region of the brain. **c** Akt proteins levels in the frontal (F _(2, 5)_ = 12.19, *p* = 0.0120) and middle (F _(2, 11)_ = 1.460, *p* = 0.2740) region of the brain. P-Akt levels in frontal (F _(2, 14)_ = 7.215, *p* = 0.0070 and middle (F _(2, 11)_ = 9.246, *p* = 0.0044) region of the brain. **d** P-Akt/Akt ratio expression in the frontal (F _(2, 7)_ = 12.5, *p* = 0.0048) and middle (F _(2, 11)_ = 19.83, p = 0.0002) region of the brain. **e** TTC representation of infarcted regions. White areas indicative of . Protein levels were normalized by the corresponding GAPDH and related total protein expressions. The values are the percentage of the corresponding relative intensity of the control (Sham). Representative western blots are presented with cropped blot panels showing target protein signals and control (GAPDH) protein signals in separate panels derived from the same gel. Graphs show mean ± SEM. * and # significant compared to sham & vehicle treated group, respectively by ANOVA and Tukey post hoc tests (*n* = 5, *p* < 0.05)

G-CSF and its receptor are expressed by neurons in the CNS, and their expression is regulated by ischemia, which points to an autocrine protective signaling mechanism [24]. G-CSF is reported to improve long-term behavioral outcomes after cortical ischemia, while also stimulating a neural progenitor recovery response in vivo. In carrying out a molecular analysis of the effect of G-CSF in global cerebral ischemia, we performed western blot to determine the presence of G-CSFR in the brain (Fig. 1a). We found the G-CSF treatment can upregulate level of G-CSFR (Fig. 1a) and G-CSF protein (Fig. 1b) in the frontal and middle region of stroke treated group with G-CSF treatment compared to vehicle treated group in BCAO.

Levels of G-CSFR expression were decreased by stroke to less than 50% relative to untreated Sham animals. G-CSF treatment can increase expression of G-CSFR by greater than 1.9 fold and 2.1 fold in the frontal brain and middle brain regions respectively relative to vehicle treated BCAO animals. Similarly, we see the effect of G-CSF treatment increases expression of G-CSF protein by 2.4 fold and 1.9 fold in frontal and middle brain regions respectively compared to vehicle treated BCAO.

Phosphorylated Akt (P-Akt) is a well-established biomarker for cell protection [33], levels of P-Akt is increased as G-CSF binds to G-CSF receptor at cell membrane [23]. We found that P-Akt, a pro-survival biomarker, is increased towards normal levels by G-CSF treatment as shown in Fig. 1c. Our data indicates that the activated form of Akt (phosphorylated Akt or P-Akt) showed a dramatic up- regulation in the frontal & middle brain region of the G-CSF-treated groups in comparison to the vehicle-treated BCAO group (Fig. 1c). Levels of P-Akt expression were decreased to less than 70% of sham and GCSF treatment increased p-Akt expression 2 fold and 1.3 fold in frontal and middle brain regions respectively relative to vehicle treated BCAO. G-CSF treatment increased ratio of P-Akt to Akt up to 200% in frontal brain and 130% in middle brain compared to sham (Fig. 1d). Besides, we wished to assess any effect the expressed G-CSF protein may have on gross brain morphology. By performing TTC on the frontal and middle brain sections, we detected a clear representation of less infarction in the brains of G-CSF mice over the BCAO only mice (Fig. 1e). Although we did not quantify infarct volume in this study, the infarct representation is supported by several evidence that G-CSF significantly reduces infarct volume in cerebral ischemia on day 4 [23, 36].

### Protective effect of G-CSF against BCAO induced endoplasmic reticulum (ER) stress

G-CSF can modulate the unfolded protein response and ER stress-induced apoptosis [22].

**Fig. 2:**
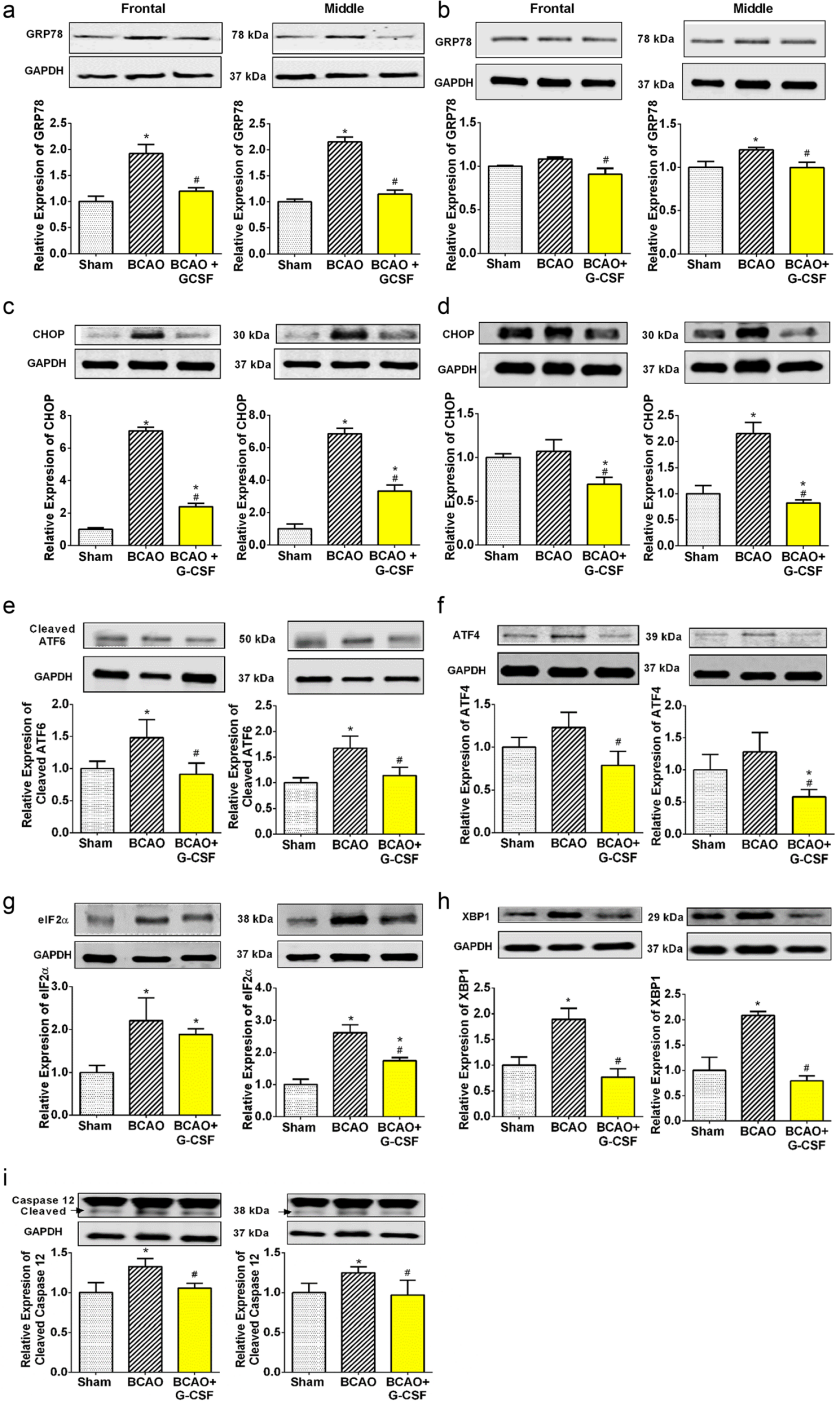
Effect of G-CSF on expression of GRP78, CHOP and ER stress proteins. **a** GRP78 levels in the frontal (F _(2, 18)_ = 9.549, *p* = 0.0015) and middle F _(2, 12)_ = 283.3, *p* < 0.0001) region of brain on day 4. **b** GRP78 levels in the frontal (F _(2, 15)_ = 4.378, *p* = 0.0318) and middle (F _(2, 14)_ = 4.787, *p* = 0.0261) region of brain on day 7. **c** CHOP levels in the ischemic frontal (F _(2, 21)_ = 10.77, *p* = 0.0006) and middle (F _(2, 11)_ = 74.31, *p* < 0.0001) region of brain on day 4. **d** Levels of CHOP in the ischemic frontal (F _(2, 14)_ = 6.101, *p* = 0.0473) and middle (F _(2, 20)_ = 26.63, p < 0.0001) region of brain on day 7. **e** Cleaved ATF6 levels in the frontal (F _(2, 27)_ = 5.464, *p* = 0.0102) and middle (F _(2, 26)_ = 6.514, *p* = 0.0051) region of brain. **f** ATF4 levels in the frontal (F _(2, 15)_ = 4.084, *p* = 0.0384) and middle (F _(2, 13)_ = 3.965, *p* = 0.0452) region of brain. **g** eIF2α levels in the ischemic frontal (F _(2, 8)_ = 5.671, *p* = 0.0293) and middle (F _(2, 11)_ = 21.24, p = 0.0002) regions of brain. **h** XBP1 levels in the frontal (F _(2, 12)_ = 10.29, *p* = 0.0025) and middle (F _(2, 7)_ = 19.53, *p* = 0.0014) region of brain. **i** Cleaved caspase-12 levels in the ischemic frontal (F _(2, 9)_ = 8.165, *p* = 0.0095) and middle (F _(2, 11)_ = 5.357, *p* = 0.0238) regions of brain. Representative western blots are presented with cropped blot panels showing target protein signals and control (GAPDH) protein signals in separate panels derived from the same gel. Graphs shows mean ± SEM. * and # significant compared to sham & vehicle treated groups, respectively by ANOVA and Tukey post hoc tests (*n* = 5, p < 0.05)

In analyzing ER stress pathways, we found that G-CSF decreased expression of GRP78 to less than 70% and less than 60% in frontal and middle regions respectively relative to vehicle treated BCAO. (Fig. 2a and b). Our data indicated that analysis on Day 4 (Fig. 2a), showed larger decrease in GRP78 expression compared to Day 7(Fig. 2b).

We examined all three ER stress signaling pathways (ATF6, PERK and IRE-1) on Day 7.

Treatment with G-CSF can decrease cleaved ATF6 level by up to 60% in frontal brain and 40% in middle brain compared to vehicle treated BCAO group (Fig. 2e). Our data showed that G-CSF treatment decreased ATF4 levels by up to 45% in frontal brain and by up to 60% in middle brain compared to vehicle treated BCAO group (Fig. 2f). Upon treatment with G-CSF, levels of eIF2α decrease by up to 25% in frontal brain and 35% in middle brain compared to vehicle treated BCAO group (Fig. 2g).

With respect to the IRE1 pathway, the results showed that XBP1 is highly expressed in the frontal & middle region of the brain in the BCAO mouse in comparison to the sham-operated group. Our data showed that G-CSF treatment decreased XBP-1 levels to less than 50 and 40% respectively relative to vehicle treated BCAO. (Fig. 2h).

Cleaved caspase − 12 protein expression was decreased in the GCSF treated group to less than 75% relative to vehicle treated BCAO (Fig. 2i). No significant change was seen in the level of caspase-12 on Day 7.

PERK, IRE-1 and ATF6 all converge on the promoter of the gene encoding the protein CHOP, which controls the BCL-2 family [22, 37]. At day 4 a significant drop was observed in CHOP to less than 30 and 50% in front and middle regions respectively relative to vehicle treated BCAO. Similarly, at day 7 there was a drop in CHOP to less than 60 and 35% in front and middle regions respectively relative to vehicle treated BCAO (Fig. 2c and d).

### Effect of G-CSF on the expression of mitochondrial stress biomarkers, OPA1 fusion protein, DRP1 fission protein and P53 by western blot analysis and immunohistochemistry

**Fig. 3:**
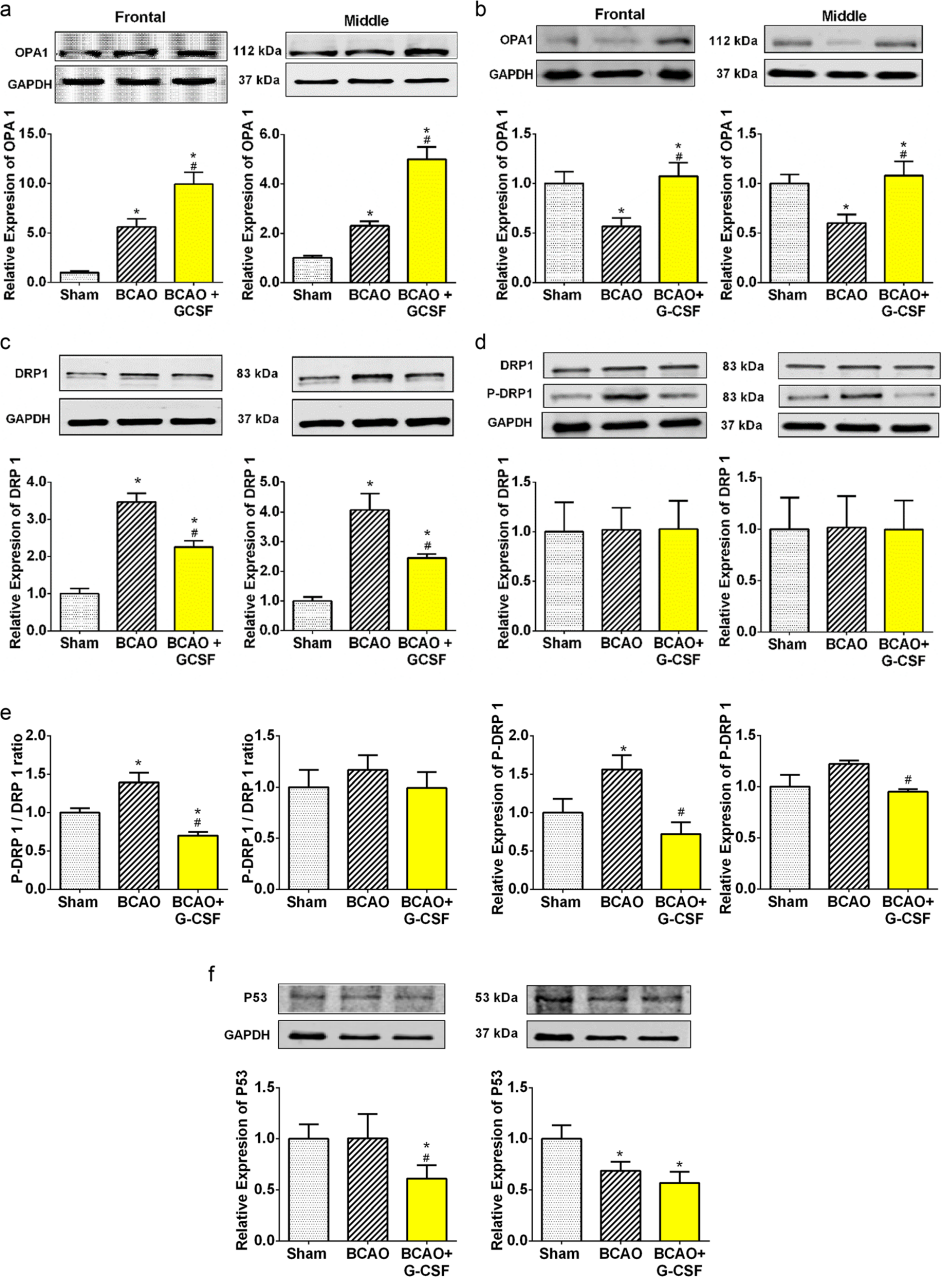
Effect of G-CSF on expression of Mitochondrial stress proteins. **a** Levels of OPA1 in the ischemic frontal (F _(2, 19)_ = 147.1, p < 0.0001) and middle (F _(2, 19)_ = 33.75, p < 0.0001) region of mouse’s brain on day 4. **b** Western blot analyses measuring levels of OPA1 in the ischemic frontal (F _(2, 28)_ = 5.366, *p* = 0.0106) and middle (F _(2, 27)_ = 4.747, *p* = 0.0171) region of mouse’s brain on day 7. **c** Levels of DRP 1 in the ischemic frontal (F _(2, 22)_ = 37.78, p < 0.0001) and middle (F _(2, 18)_ = 16.49, p < 0.0001) region of mouse’s brain on day 4. **d** Levels of DRP 1 in the ischemic frontal (F _(2, 21)_ = 0.002564, *p* = 0.9974) & middle (F _(2, 22)_ = 0.001202, *p* = 0.9988) and P-DRP1 levels in the ischemic frontal (F _(2, 14)_ = 5.692, *p* = 0.0155) & middle (F _(2, 11)_ = 4.043, *p* = 0.0483) regions of mouse’s brain on day 7. **e** Western blot analyses indicating ratio of levels of P-DRP1/DRP1 in the ischemic frontal (F _(2, 15)_ = 16.50, p = 0.0002) and middle (F _(2, 12)_ = 0.4053, *p* = 0.6755) regions of mouse’s brain on day 7. **f** P53 expression levels in the frontal (F _(2, 13)_ = 4.803, *p* = 0.0274) and middle (F _(2, 9)_ = 6.150, *p* = 0.0207 Vs Sham); (F _(2, 10)_ = 3.801, *p* = 0.0592 vs BCAO) region of brain. Representative western blots are presented with cropped blot panels showing target protein signals and control (GAPDH) protein signals in separate panels derived from the same gel. Graphs show mean ± SEM. * and # significant compared to sham & vehicle treated groups, respectively by ANOVA and Tukey post hoc tests (n = 5, p < 0.05)

In addition to ER stress, we have also examined the effect of G-CSF on mitochondrial function in BCAO mice as determined by the level of mitochondrial stress marker, DRP1 and the level of mitochondrial pro-survival marker, OPA1. Both OPA1 and DRP1 are markers of mitochondrial dynamics [38, 39]. During mitochondrial dysfunction, the mitochondria will undergo fission (fragmentation), resulting in an increase in DRP1 (Fig. 3g) [40]. We found that BCAO caused a significant increase of the mitochondrial marker DRP1 at day 4 by greater than 3.1 fold and 3.3 fold respectively in front and middle regions relative to sham (Fig. 3c). G-CSF treatment reduced the DRP1 level at Day 4 to less than 60 and 50% respectively in frontal and middle regions relative to vehicle treated BCAO (Fig. 3c). On day 7, there is no significant change in DRP1 levels. P-DRP1 was increased by greater than 1.6 fold and 1.2 fold in front and middle regions respectively on day 7 relative to sham and with BCAO, P-DRP1 was decreased to less than 45 and 70% by G-CSF treatment relative to vehicle treated BCAO (Fig. 3d). On the other hand, the marker of enhanced mitochondrial integrity, OPA1 is strongly elevated by G-CSF treatment at day 4 by greater than 1.8 fold and 2.2 fold in front and middle regions respectively relative to vehicle treated BCAO (Fig. 3a). At day 7 OPA1 was similarly increased with G-CSF treatment by greater than 2 fold and 1.9 fold in front and middle regions respectively relative to vehicle treated BCAO (Fig. 3b). P53 is well known for its role in neuronal cell death. Multiple studies have been conducted involving the P53 signaling pathway in apoptosis after cerebral ischemia [41].

Upon mitochondrial stress, P53 translocates from the cytoplasm to the mitochondria where it acts in conjunction with BH3 only proteins (such as BAX and PUMA) causing apoptosis [42]. We found that P53 level was significantly reduced to less than 50%, in the frontal brain region of the BCAO group treated with G-CSF compared to the vehicle treated BCAO group (Fig. 3f). A significant increase of the presence of DRP1 was observed in the middle brain region of the cerebrum of BCAO/Vehicle mice compared to the midbrain region of the cerebrum of BCAO/G-CSF mice (Additional file 1: Figure S1a, 1b & 1c). This finding suggests mitochondrial fission is taking place secondary to the BCAO leading to apoptosis. OPA1 is involved in mitochondrial fusion and aids in neuroprotection. OPA1 signaling was detected at a higher level in BCAO/G-CSF treated than in the middle brain region of the cerebrum of BCAO/Vehicle and absent in sham animals (Additional file 1: Figure S1d,1e & 1f).

In this study, pro-apoptotic P53 (Additional file 1: Figure S1 g, 1 h & 1i) was identified to be present in the middle brain of the BCAO/Vehicle but absent in the middle brain of BCAO/G-CSF and Sham mice suggesting G-CSF decreases apoptotic protein signaling (DRP1 and P53) and increases pro-survival signaling (OPA1).

### Effect of G-CSF on the expression Beclin 1, marker of autophagy

**Fig. 4:**
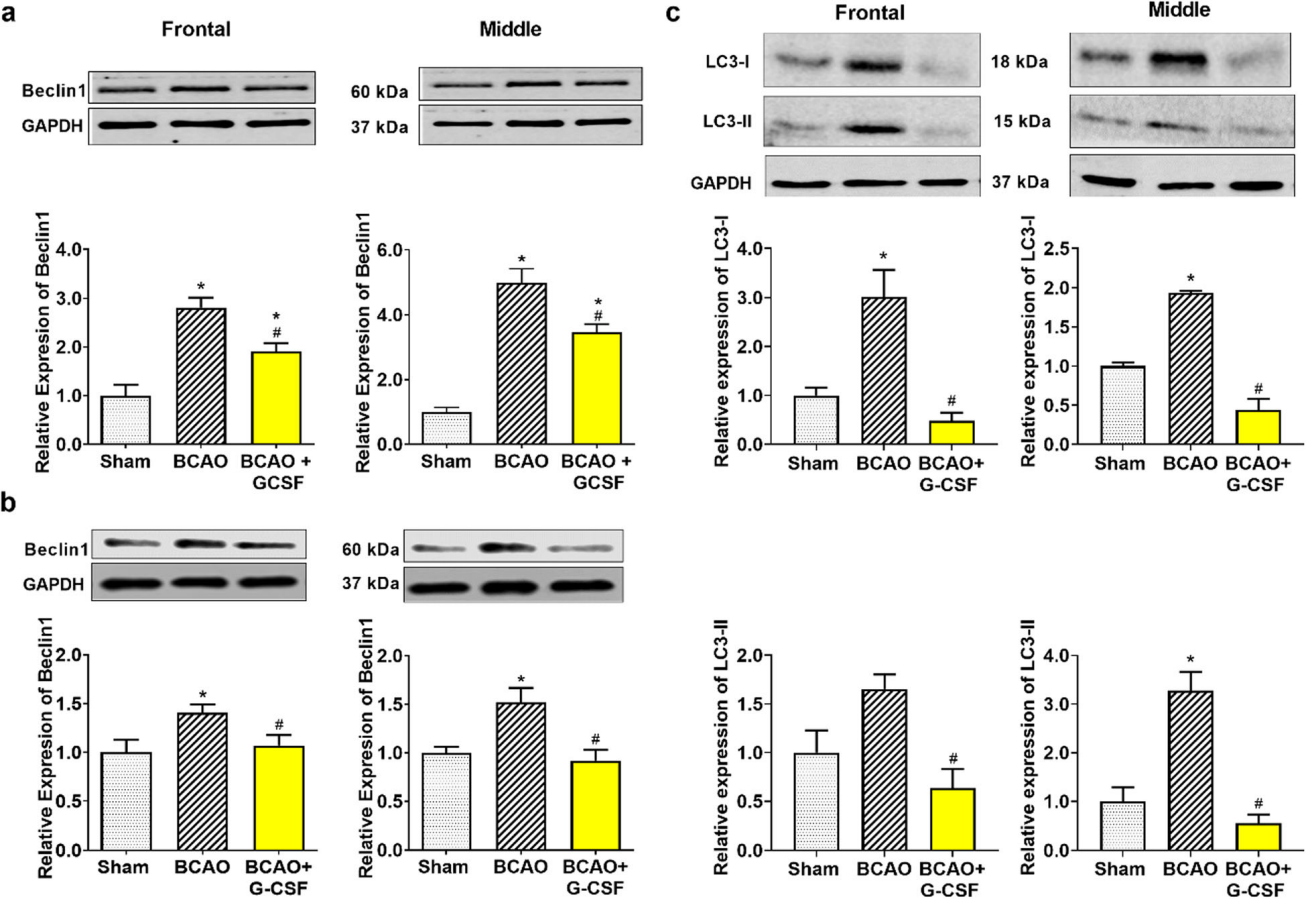
Effect of G-CSF therapy on Beclin1, LC3-I & II expression in BCAO mice. **a** Levels of Beclin1 in the ischemic frontal (F _(2, 9)_ = 20.08, *p* = 0.0005) and middle (F _(2, 12)_ = 47.52, p < 0.0001) region of mouse’s brain on day 4. **b** Levels of Beclin1 in the ischemic frontal (F _(2, 7)_ = 7.406, *p* = 0.0187) and middle (F _(2, 12)_ = 4.020, *p* = 0.0461) region of mouse’s brain on day 7. **c** Levels of LC3-I in the ischemic frontal (F _(2, 21)_ = 17.81, p < 0.0001) and middle (F _(2, 15)_ = 35.92, p < 0.0001) and LC3-II levels in ischemic frontal (F _(2, 18)_ = 6.711, *p* = 0.0066) and middle (F _(2, 24)_ = 26.01, p < 0.0001) region of mouse’s brain on day 7. Representative western blots are presented with cropped blot panels showing target protein signals and control (GAPDH) protein signals in separate panels derived from the same gel. Graphs show mean ± SEM. * and # significant compared to sham & vehicle treated groups, respectively by ANOVA and Tukey post hoc tests (n = 5, p < 0.05)

Beclin 1, an autophagy maker [8, 10], shows a reducing trend for the G-CSF treatment in both the frontal and middle sections of the brain compared to vehicle treated BCAO group on day 4 as shown in Fig. 4a. Our data indicates that G-CSF reduces the need for the cell to digest/destroy itself (cellular self-digestion occurs when a cell is stressed). Similar results were obtained at 7 days after BCAO as shown in Fig. 4b. Microtubule-associated proteins (MAP)-light chain 3 β (LC3-II) is essential for autophagy and is associated with autophagosome membranes after processing, MAP LC3α (LC3-I) is involved in the formation of autophagosomal vacuoles and is localized to the intracytoplasmic membrane. Both MAPs are expressed primarily in heart, testis, brain and skeletal muscle [11, 43, 44]. G-CSF treatment significantly decreased expression of LC3 lipidation, notably LC3-I, to less than 20 and 25% in frontal and middle brain regions respectively relative to vehicle treated BCAO (Fig. 4c, upper panel). Similarly, G-CSF decreased levels of LC3-II protein to less than 40 and 20% in frontal and middle brain regions respectively relative to vehicle treated BCAO. (Fig. 4c, lower panel).

### Effect of G-CSF on apoptosis by down-regulation of apoptotic markers

Expression of Bcl-2, an anti-apoptotic regulator, is induced in the brain by ischemia consistent with the potential role of this protein as part of endogenous neuro-protective mechanism [30, 33].

**Fig. 5:**
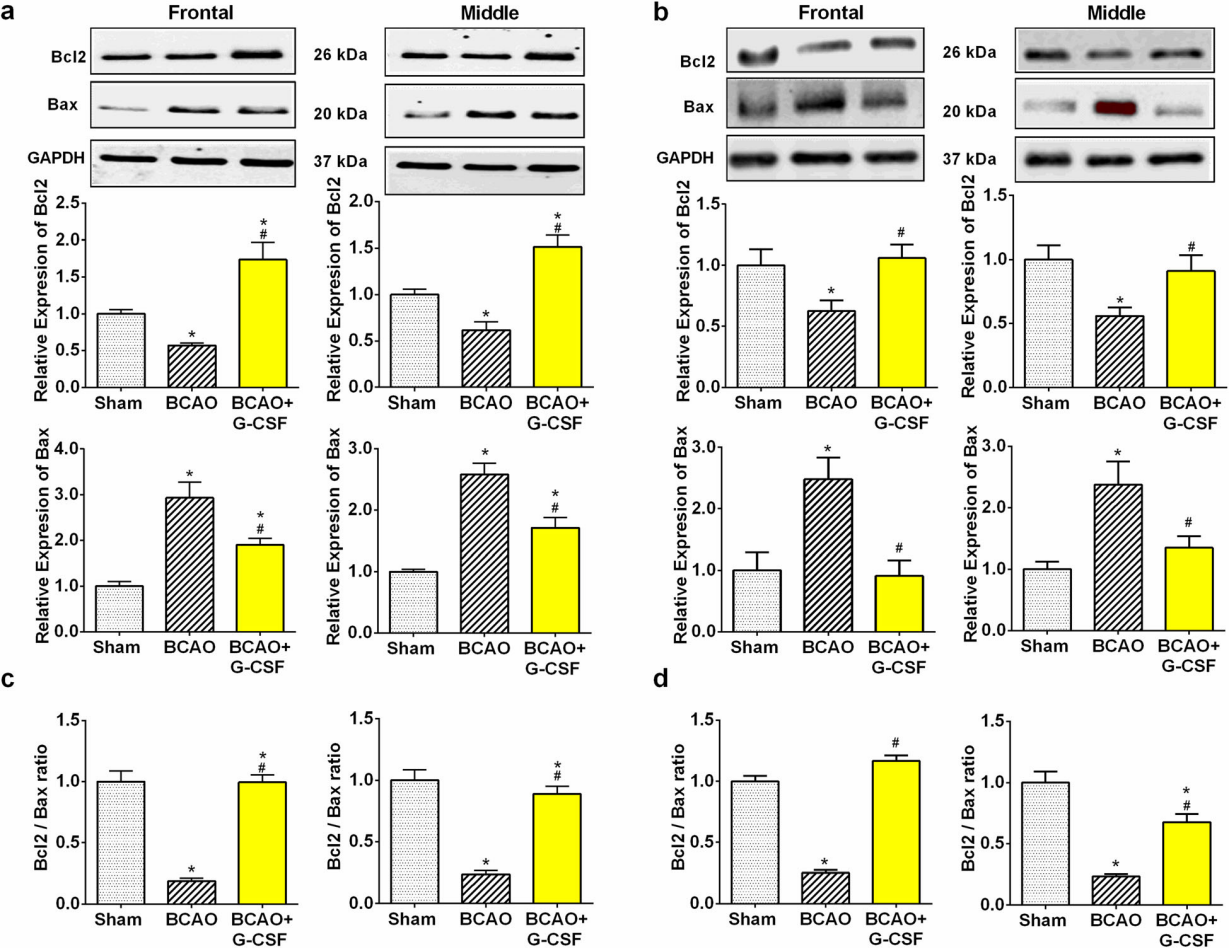
Effect of G-CSF therapy on Bcl- 2 and Bax expression in BCAO mice. **a** Bcl-2 levels in the frontal (F _(2, 9)_ = 17.52, *p* = 0.0008) and middle (F _(2, 9)_ = 22.5, *p* = 0.0003) region of brain on day 4 and, Bax levels in the frontal (F _(2, 9)_ = 19.01, p = 0.0006) and middle (F _(2, 9)_ = 29.96, *p* = 0.0001) region of brain on day 4. **b** Bcl-2 levels in the frontal (F _(2, 27)_ = 4.571, *p* = 0.0195) and middle (F _(2, 28)_ = 5.352, *p* = 0.0108) region of brain on day 7. And, Bax levels in the frontal (F _(2, 16)_ = 7.959, *p* = 0.0040) and middle (F _(2, 26)_ = 5.817, *p* = 0.0082) region of brain on day 7. **c** Bcl-2/Bax ratio expression in the frontal (F _(2, 9)_ = 54.70, p < 0.0001) and middle (F _(2, 9)_ = 42.32, p < 0.0001) region of brain on day 4. **d** Bcl-2/Bax ratio expression in the frontal (F _(2, 16)_ = 114.6, p < 0.0001) and middle (F _(2, 27)_ = 43.12, p < 0.0001) region of brain on day 7. Representative western blots are presented with cropped blot panels showing target protein signals and control (GAPDH) protein signals in separate panels derived from the same gel. Graphs show mean ± SEM. * and # significant compared to sham & vehicle treated groups, respectively by ANOVA and Tukey post hoc tests (n = 5, p < 0.05)

**Fig. 6:**
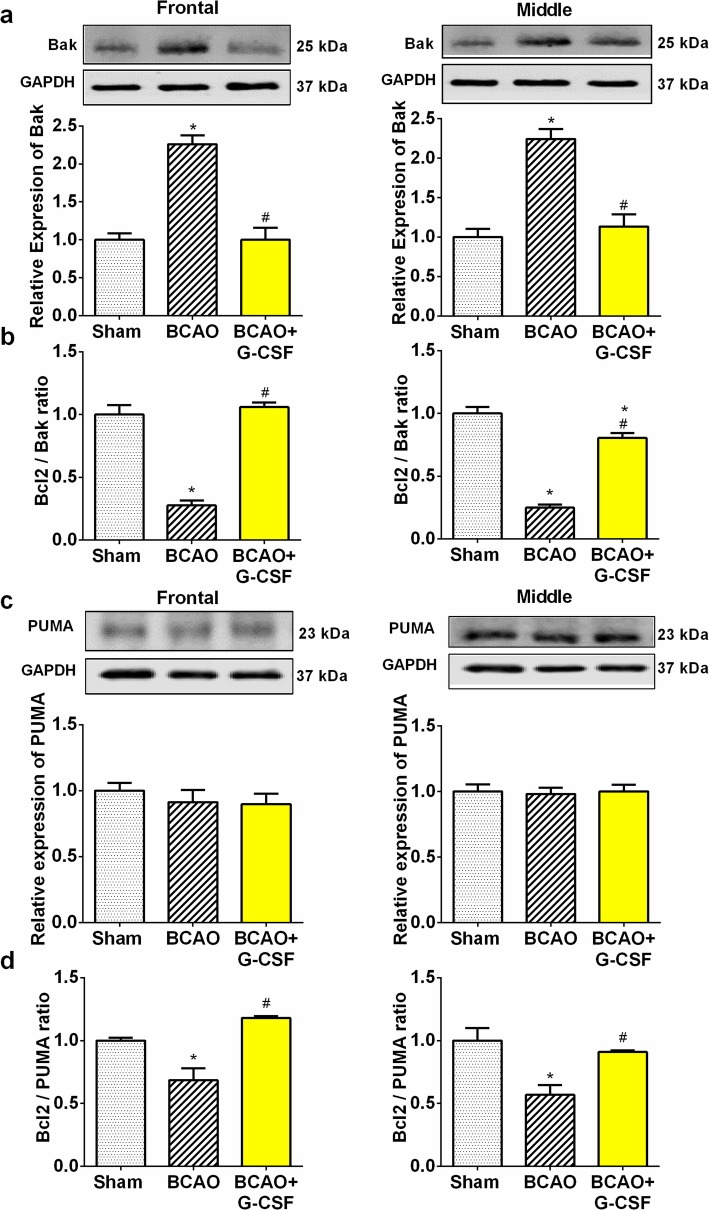
Effect of G-CSF on expression of Bak and PUMA in BCAO stroke model. **a** Bak expression in the frontal (F _(2, 9)_ = 34.77, p < 0.0001) and middle (F _(2, 12)_ = 20.85, p = 0.0001) region of BCAO brain were analyzed by Western blot. **b** Bcl-2/Bak ratio expression in the frontal (F _(2, 9)_ = 67.67, p < 0.0001) and middle (F _(2, 12)_ = 71.9, p < 0.0001) region of BCAO in G-CSF treated group markedly increased versus vehicle treated BCAO group. Treatment of G-CSF significantly decreased Bak level (a) in comparison to the vehicle treated BCAO group. **c** Western blot analyses showed that G-CSF treatment group has no significant effect on PUMA expression in the ischemic frontal (F _(2, 11)_ = 0.6084, *p* = 0.5616) and middle (F _(2, 10)_ = 0.04798, *p* = 0.9534) regions of mouse’s brain on day 7. **d** Bcl-2/PUMA ratio expression in the frontal (F _(2, 11)_ = 22.07, p = 0.0001) and middle (F _(2, 12)_ = 12.26, *p* = 0.0013) region of BCAO in G-CSF treated group markedly increased versus vehicle treated BCAO group. Representative western blots are presented with cropped blot panels showing target protein signals and control (GAPDH) protein signals in separate panels derived from the same gel. Values in the graphs represent mean ± SEM. * and # significant compared to sham & vehicle treated groups, respectively by ANOVA and Tukey post hoc tests (n = 5, p < 0.05)

Our results demonstrate that G-CSF can upregulate anti apoptotic protein Bcl2 (Fig. 5a and b) and down-regulate proapoptotic proteins Bax and Bak in both in the frontal & middle region of BCAO brain (Fig. 5a and b & Fig. 6a). On the other hand, measurement of anti-apoptotic protein BCL-2 showed an increase in the ratios of BCL-2/Bax (Fig. 5c and d), and Bcl2/Bak in the frontal and middle region of the G-CSF treated versus vehicle-treated group of BCAO animals (Fig. 6b).

Notably in our analysis while PUMA a pro-apoptotic activator was not differentially regulated between conditions (Fig. 6c), measurement of the ratio of pro-survival Bcl-2 to pro-death PUMA (Bcl-2/PUMA) on day 7 demonstrated up-regulation of 45 and 35% respectively, following G-CSF treatment in the ischemic frontal & middle region of BCAO brain (Fig. 6d).

### Behavioral tests

**Fig. 7:**
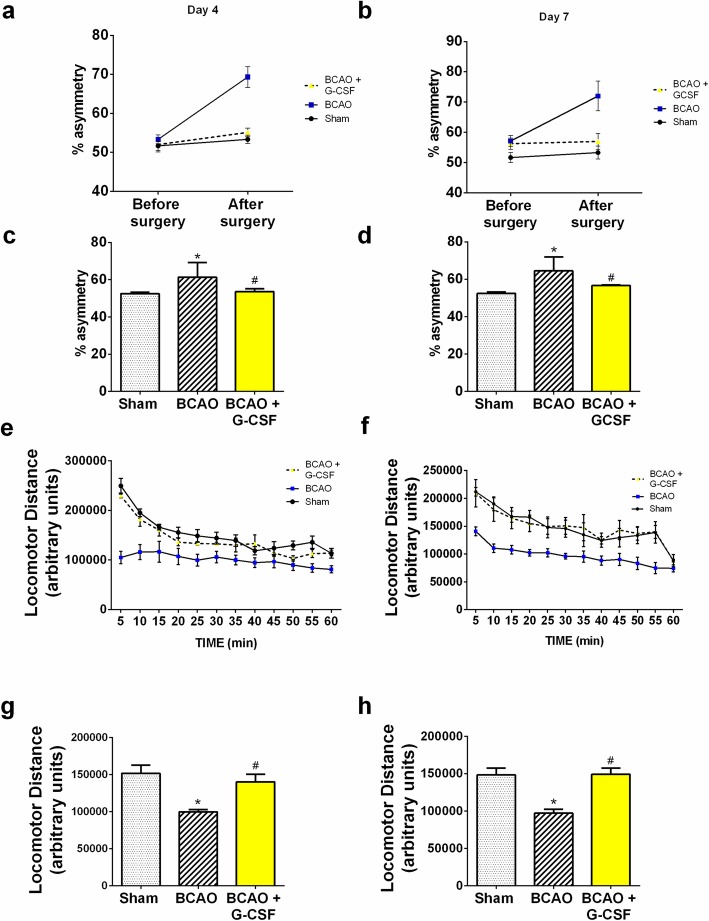
Effect of G-CSF protein on Behavioral test demonstrated in the a) Corner Test behavioral assay; b) locomotor activity test. **a, b** Percentage asymmetry is observed as a mouse enters a 30^0^ corner and either turns left or right. In the above graph **a, b** percent asymmetry is compared before and after BCAO for sham, BCAO/Vehicle, and BCAO/G-CSF (*n* = 6). It is observed that mice treated with G-CSF after BCAO had a significant better percent asymmetry than those of BCAO/Vehicle and like the percent asymmetry of Sham mice on day 4 (F _(5, 82)_ = 17.69, p < 0.0001) and day 7 (F _(5, 32)_ = 5.902, p = 0.0006). **c, d** Average results (n = 6) of Corner test densitometric scanning are presented. (All data are presented as mean +/− SEM, #/* *p* < 0.05. # statistical significance between groups Sham and BCAO + Vehicle; *statistical significance between groups BCAO + Vehicle and BCAO + G-CSF). **e, f** Neuroprotection effect of G-CSF protein therapy in BCAO stroke mouse model using locomotor activity test. Locomotor activity of BCAO mice with and without G-CSF treatment were done after 4 days. Results show increased amount of activity with administration of G-CSF on day 4 (F _(1.179, 12.97)_ = 26.12, p = 0.0001) and day 7 (F _(1.259, 13.85)_ = 105.1, p < 0.0001). **g, h** Average results (n = 5) of locomotor test densitometric scanning are presented. The summary of locomotor data is provided in expressed in mean +/− SEM where n = 5 for Sham, BCAO and BCAO + G-CSF. **a, c, e** and **g** represent day 4; **b, d, f and h** represent day 7. Data shows animals treated with G-CSF have activity consistent with sham animals

Unlike mice with CNS damage, it has been shown that on encountering a corner normal animals run into a corner and naturally rear forward and upward, then turn back, in either direction, to face away from the corner and toward the open end of the setup (the corner test) [26]. This determines an animal’s asymmetric direction of turning when encountering a 30^0^ corner which is used as an indicator of brain injury. We used an experimental corner set up composed of two boards (30 × 20 × 1 cm3) arranged to form a 30^0^ corner. Normal mice were found to show a rate of 50 ± 8% (symmetric and without bias) before BCAO-30 min surgery. At 4 days after BCAO we observed a significant increase in asymmetric turning (~ 90% to one side) in BCAO mice. When facing a 30° corner. BCAO mice without treatment showed significant asymmetric turning (~ 90%) beginning one day after the procedure and persisting for at least four days (Fig. 7a and b). Mice with treatment (BCAO + G-CSF) exhibited behavior not significant differently from that seen in the sham-operated mice (Fig. 7c and d).

To test whether motor dysfunction might arise with BCAO, the locomotor activity of mice was measured on a force-plate actometer [27, 28, 45]. We observed no significant difference in behavior between sham group and BCAO with G-CSF treated group (Fig. 7e and f). The travel distances and the number of Sham group, BCAO with G-CSF group were statistically significantly different compared to BCAO with vehicle group on day 4 (Fig. 7g distance: Sham: 151 ± 11.75 m; BCAO with vehicle: 100 ± 3.25 m; BCAO with G-CSF: 140 ± 10.05, *n* = 5, *p* < 0.05) and day 7 (Fig. 7h distance: Sham: 149 ± 9.35 m; BCAO with vehicle: 97 ± 5.15 m; BCAO with G-CSF: 149 ± 8.45, n = 5, p < 0.05).

## Discussion

In recent years, several studies including ours have revealed that G-CSF as an endogenous growth factor and immune system modulator factor [46] is beneficial in models of neurological disorders such as stroke and traumatic brain injury [23, 47] Although the anti-apoptotic activity of G-CSF is reported in global cerebral ischemia, this mechanism is still not fully explored. We induced transient global ischemia/reperfusion injury in male Swiss Webster mice by using the carotid artery clamping method to occlude the CCA for 30 mins. The ischemia produced provided a suitable reduction in regional blood flow < 51% drop of baseline and > 93% return to baseline during reperfusion (data not shown).

To investigate the efficacy of G-CSF beyond the usual 4 h (TPA) treatment window for global ischemia, the initial dose of G- CSF was administered 24 h post-BCAO. This initial dose was then followed by a single application, of the same dose, for another 3 days resulting in a total of 4 days of G-CSF administration. Our previous paper reported that the initial administration of G-CSF (50 μg/kg body weight. s.c.) after 24 h post-MCAO in rat has beneficial effects on stroke with reduction of infarct volume [23].

We have demonstrated that G-CSF was protective against ER stress elicited apoptosis by downregulating ER stress-induced apoptotic CHOP and downregulating the expression levels of ATF4, XBP1, eIF2α and cleaved ATF6, the down- stream targets in the ER stress sensor pathways: PERK, IRE1 and ATF6, respectively (Fig. 2j). G-CSF has also a significant effect on the ER stress associated protein; cleaved caspase-12. G-CSF also attenuated the GRP78 and Akt in both the frontal and middle area of the ischemic brain as well as increasing Bcl-2 expression while reducing Bax and Bak expression. G-CSF has effect on Autophagy marker Beclin-1 and mitochondrial stress proteins which is consistent with a decrease in apoptotic protein signaling (DRP1 and P53) & and an increase in apoptotic inhibitor (OPA1). On day 4 and day 7 after occlusion, G-CSGF treatment improves the neurological deficit. To our knowledge this report on the protective effect of G-CSF against autophagy and mitochondrial stress is a unique and novel finding.

Caspase-12 participates in inflammatory pathways and is a key molecule related to ER stress-induced apoptosis signaling pathways in neuronal death following ischemia/reperfusion [48]. Notably, our results reveal that BCAO can increase the caspase-12 cleavage after 7 days in the frontal & middle brain. However, we showed that G-CSF treatment decreased this cleavage to less than 20% in frontal brain and 25% in middle brain compare to non-treatment groups. Reorganization of calcium homeostasis by G-CSF may be one of the reasons for downregulation of caspase-12 and preventing its cleavage.

GRP78 in the ER is used as a marker for the unfolding protein response (UPR). GRP78 has several functions in the cell including protein folding in the ER, the UPR and inhibition of apoptosis [22, 37]. GRP78 displayed a significant increase in MCAO 4 days after reperfusion [23, 30]. However, in these current experiments on BCAO, G-CSF decreased the expression of GRP78 in comparison to the vehicle-treated group.

The UPR disconnects GRP78 from its sensors (ATF6, PERK and IRE-1) inside the ER lumen in response to buildup of unfolded proteins. Our previous study confirmed that G-CSF has beneficial effects on the protection against ER stress in the penumbra and core of the MCAO infarct [23]. Following detachment of GRP78, PERK is turned on and then phosphorylates a subunit of eIF2a [37] which in turn inhibits general cap-dependent translation thus decreasing further the buildup of proteins within the ER lumen [22, 37]. However, PERK activates two down-stream proteins in the PERK pathway of ER stress (namely eIF2-alpha and ATF4). We found that in the BCAO model of stroke, there was a clear increase in eIF2α, and ATF4 expression, indicating that the PERK pathway is activated in BCAO models. Our data showed that the PERK pathway is strongly inhibited in the Frontal and Middle brain samples following use of G-CSF in BCAO.

ATF6 signaling is mostly pro-survival with little evidence linking it to cell death [37, 49]. We showed in our experiment that the ratio of cleaved ATF6 to full-length ATF6 indicates that G-CSF decreased ATF6 cleavage in the Frontal and Middle brain samples using the BCAO stroke model.

In ER stress IRE1a signals to synthesize the mRNA for the transcription factor X box binding protein (XBP1). XBP1 is responsible for regulating a specific subset of UPR target genes, involved in folding and ER-associated degradation (ERAD) [30]. IRE1a also interacts with adaptor proteins in the cytosol and initiates signaling pathways known as alarm pathways (including JNK, Ask1, and NF-kappaB) resulting in triggering of autophagy and apoptosis [23, 37].

Under severe stress, the UPR pathways signal for apoptosis via the key transcription factor C/EBP homologous protein (CHOP), which differentially regulates transcription of genes encoding both pro- and anti- apoptotic Bcl-2 family members respectively (Fig. 2j). Our data showed that CHOP that was upregulated in the frontal and middle brain samples of the BCAO model and demonstrated a significant decrease in levels in the frontal and middle brain samples of G-CSF treated groups.

Several findings have indicated that activated Akt (P-Akt) promotes neuroprotection during cerebral ischemia [30]. We demonstrated that G-CSF activates Akt (Fig. 1e), thereby eliciting neuroprotection in BCAO after 4 days of treatment. Use of G-CSF caused a markedly increased level of P-Akt expression in the frontal and middle brain samples of the G-CSF-treated group. The phosphorylation of Akt (P-Akt) was increased in neuronal cells, and phospho-Akt colocalized with Beclin-1 in the neonatal ischemic model [2, 50] Knock down of Beclin-1 with small interfering RNA (siRNA) was shown to inhibit neuronal autophagy and protect against hypoxia induced excitotoxicity in rat [50]. We found that BCAO activates Beclin 1, an autophagy marker on day 4 and day 7 and LC3 lipidation on day 7 in the vehicle treated group of BCAO. G-CSF treatment markedly decreased Beclin 1 and LC3 lipidation (LC3-I and LC3-II) in frontal and middle brain treated with G-CSF. An Akt inhibitor significantly inhibited the autophagy process by reducing Beclin-1, LC3-I and LC3-II expression and resulted in switching the mechanism of cell death from apoptosis to necrosis [11, 50–52]. Activation of the Akt pathway, as well as Akt-mediated Bcl-2 phosphorylation and Bcl-2/Beclin-1 complex disruption, has been shown to occur [53]. The Akt serine/threonine kinases are crucial moderators of cell survival in response to hypoxic injury [37]. A number of pro-apoptotic proteins have been characterized as direct Akt substrates, and those include p53, Bax and Bak which are suppressed upon phosphorylation by Akt [42, 54]. P- Akt also has been shown to induce some anti-apoptotic markers, such as BCL-2 and mTOR [37]. Our data on the proapoptotic markers Bax and Bak and the anti-apoptotic marker BCL-2 support the role of P-Akt up-regulation in protecting cells in the G-CSF treated group.

The mitochondrion appears to be condensed at the point of contact with the ER, pointing to an essential role for the ER–mitochondria association in the initiation of mitochondrial fission [55]. Mitochondrial fission and fusion are integrally involved in cell division and differentiation [56, 57]. More recently, mitochondrial fission has been shown to be involved in synaptic and spine plasticity in neurons [7]. Dynamin-related protein (DRP1) localizes mostly to the cytoplasm and is employed in mitochondrial function to control mitochondrial fission [2]. Mitochondrial fission is boosted by elevated DRP1 and Fis1, and by decreased OPA1 and Mfn2 expression [5]. Two important markers of mitochondrial dynamics are OPA1 and DRP1 [10]. OPA1 is a mitochondrial fusion protein while DRP1 is a mitochondrial fission protein. During mitochondrial dysfunction, the mitochondria will undergo fission (fragmentation), resulting in an increase in DRP1(Fig. 3g) [6, 39]. We have demonstrated neuro-protection of G-CSF gene therapy in the BCAO mouse stroke model as shown by a decrease of DRP1, a marker of mitochondrial stress in frontal and middle brain in the G-CSF treated group. It was reported that an increase in the expression of OPA1 acts to alleviate brain edema in cerebral ischemic injury [50]. Our data showed that the OPA1 is clearly increased in the Frontal and Middle part of brain following use of G-CSF in BCAO. This increase in the expression of the mitochondrial fusion protein (Fig. 3g); OPA1 using G-CSF treatment in BCAO, indicates reduced mitochondrial fission, thus facilitating conservation of mitochondria.

Mitochondrial p53 is known to form inhibitory complexes with pro-survival Bcl-XL and Bcl-2 proteins, resulting in cytochrome c release from mitochondria (Fig. 3g) [2, 41]. In addition, certain p53-regulated Bcl-2 homology 3 (BH-3) only proteins, such as PUMA, Noxa, and Bid (BH-3 interacting domain death agonist), might also play vital roles in neuronal apoptosis [2, 41]. We found that levels of P53 are decreased with treatment of G-CSF compared to the vehicle treated group in BCAO. This evidence suggests that p53 can promote to apoptosis by direct signaling at the level of the mitochondria. We showed in our experiment that the ratio of Bcl2 to PUMA increased in response to G-CSF in the Frontal and Middle brain regions in the BCAO stroke model.

With G-CSF treatment, frontal and middle brain showed greater Bcl-2 expression and lower Bax/Bak expression compared to the vehicle treated BCAO group. Bcl-2 is known to play a key role in regulating neuronal survival [2]. Bcl-2-associated X protein (Bax) is also required for oxidative stress induced cell death and PUMA plays a major role in regulating Bax/Bak activation and neuronal apoptosis [33, 58]. Hence G-CSF exerts its neuroprotective role by inhibiting apoptosis via increased Bcl-2 expression and decreased Bax/Bak/PUMA expression in the frontal and middle brain.

Our results support the theory that G-CSF reduces neurological deficits that occur in the first few days after cerebral ischemia. In our previous investigation, we demonstrated that G-CSF could markedly reduce the neuro-score and volume of the lesion in MCAO after 4 days [23]. We found here that BCAO mice subjected to BCAO and receiving G-CSF protein showed significantly less asymmetric turning than BCAO mice with no G-CSF. In our behavioral assays, G-CSF elicits increased locomotor sensitization and this finding was reflected in a greater activity in the locomotor activity test, demonstrating neuroprotection of G-CSF.

## Conclusion

**Fig. 8:**
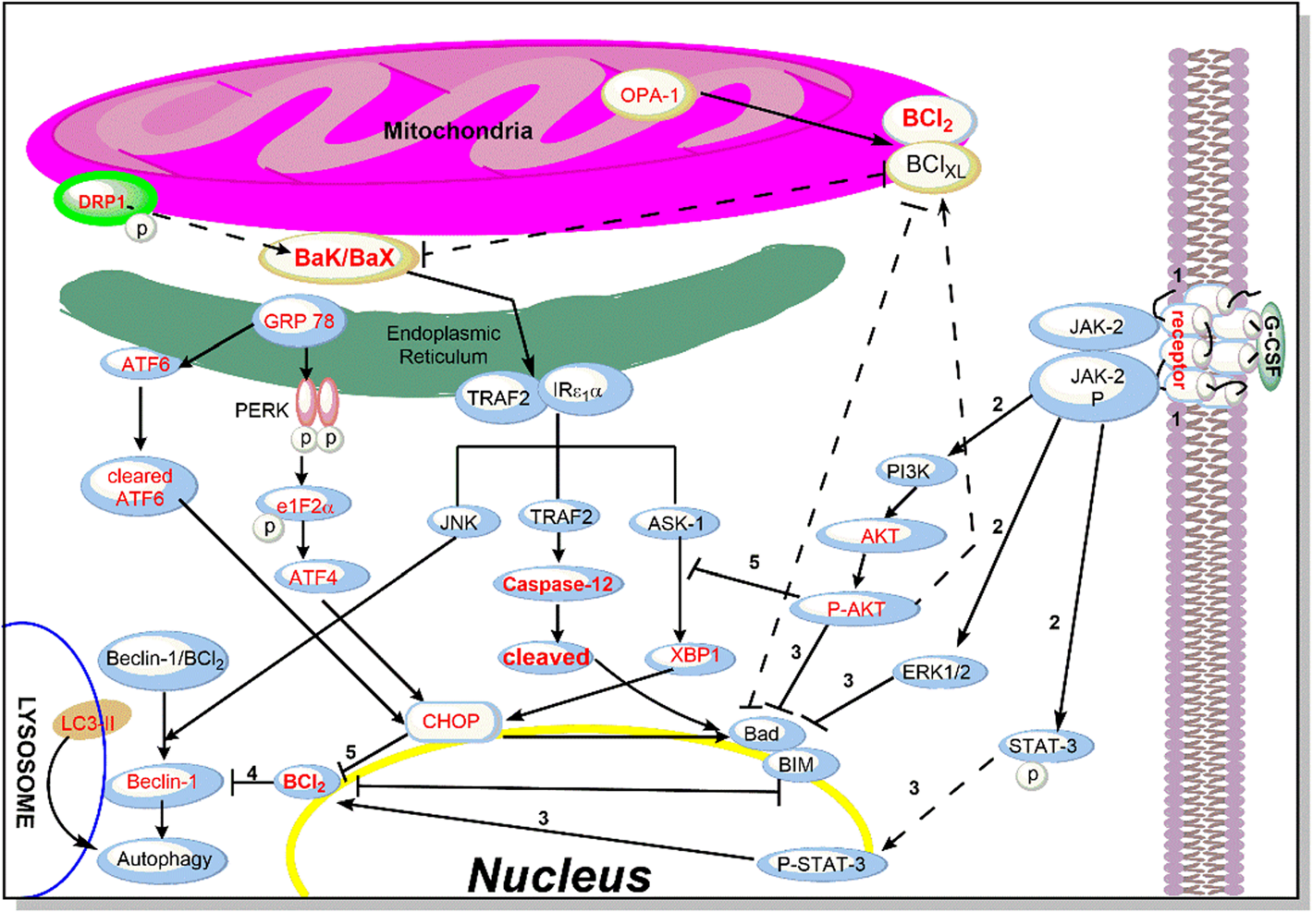
Schematic representation of G-CSF mechanism of action in BCAO mouse model. *1* – G-CSF binds to G-CSF receptors resulting in phosphorylation and activation of JAK2; *2* – Activated JAK2 then activates four transduction pathways including STAT3, PI3K, ERK1/2 and ERK5; *3* – Activated STAT3 is then translocated into the nucleus and turns on the transcription of anti-apoptotic proteins, Bcl-2 and Bcl-XL whereas activated PI3K and ERK1/2 inhibit the pro-apoptotic protein, BAD, resulting in the protection of mitochondria; *4* – Increase level of Bcl2 which combines with Beclin 1 and forms complex that inhibits autophagy activation; *5* – Activated PI3K further activate Akt/p-Akt which in turn inhibits Ask-1, one of the proteins involved in ER stress pathways resulting in decreased level of CHOP and inactivation of the downstream signaling molecules involved in apoptosis including BAD and Bim

In conclusion, the physiological events leading from activation of G-CSF receptors to neuronal protection against ischemia-induced brain injury can be described as follows (Fig. 8): Step 1 – G-CSF binds to G-CSF receptors resulting in phosphorylation and activation of JAK2; Step 2 – Activated JAK2 then activates four transduction pathways including STAT3, PI3K, ERK1/2 and ERK5; Step 3 – Activated STAT3 is then translocated into the nucleus and turns on the transcription of anti-apoptotic proteins, Bcl-2 and Bcl-XL whereas activated PI3K and ERK1/2 inhibit the pro-apoptotic protein, BAD, resulting in the protection of mitochondria; Step 4 – Increase level of Bcl2 which combines with Beclin 1 and forms complex that inhibits autophagy activation; Step 5 – Activated PI3K further activate Akt/p-Akt which in turn inhibits Ask-1, one of the proteins involved in ER stress pathways resulting in decreased level of CHOP and inactivation of the downstream signaling molecules involved in apoptosis including BAD and Bim. This proposed mode of action of G-CSF against ischemia-induced brain injury in the BCAO model through activation of G-CSF receptor leading to suppression of ER stress, mitochondrial stress, and autophagy resulting in reduced apoptosis and cell death as depicted in Fig. 8. Is supported from the following observations: Firstly, G-CSF treatment showed significant inhibition of apoptosis by activation of the PERK, ATF6 and IRE-1 pathways. We showed upregulation of Akt phosphorylation which can inhibit ischemia- induced apoptosis [59], attenuate ER stress [60] and lessen mitochondrial stress [61]. Secondly, administration of G-CSF showed that an increase in Beclin 1, LC3 I & II, CHOP and Bax was prevented in the frontal and middle brain of BCAO stroke model indicating that G-CSF can decrease autophagy (down regulation of Beclin 1) and apoptosis both in mitochondrial Ca2 + −induced apoptosis (up-regulation of BCL-2/Bax and down-regulation of Bak) and ER- induced apoptosis (down-regulation of CHOP). These data support the hypothesis that G-CSF is one of the few growth factors that can decrease infarction by decreasing ER stress and mitochondrial stress in the mouse BCAO stroke model while improving behavioral performance as reflected in the locomotor test. In this study we have provided new insights into G-CSF induced protection as it relates to ER stress and mitochondrial stress activated apoptosis in experimental global stoke. Still more experiments are needed to reveal the complete mechanisms by which G-CSF retains the ER and mitochondrial homeostasis during experimental global stroke.

## Supplementary information

**Additional file 1 Table S1.** List of antibodies used for western blotting and immunohistochemistry. **Fig. S1.** Neuroprotective effect of G-CSF Protein as determined in immunohistochemistry protein signal (a) DRP1 (b) OPA1 (c) P53. Analysis was conducted comparing of the protein signals seen within the middle region of the cerebrum in mice brain using the Immunohistochemistry technique. **a, b, c** The signal of DRP1 protein was detected to be more frequent/stronger in the vehicle animals and least in the animal treated with G-CSF after 30-min BCAO. Sham animal is observed to be in-between. **d, e, f** The signal of OPA1 proteins are detected more frequent in animals treated with G-CSF in comparison to vehicle animals after 30-min BCAO. Sham animals are seen to have no OPA1 protein signal. **g, h, i** P53 protein signaling is detected more frequent in vehicle animals in comparison to animals treated with G-CSF after 30-min BCAO. Sham animals are seen to have no P53 protein signal. For all images, nuclei were counterstained with DAPI. DRP1, OPA 1 and Neu-N protein are labeled in red, P53 is labeled in green. **a, d, g** represents Sham; **b, e, h** represents BCAO and **c, f, i** represent BCAO + G-CSF. Scale bar = 20 μm (microns). (*n* = 3).

## Data Availability

The datasets used and/or analyzed during the current study are available from the corresponding author on reasonable request.

## Abbreviations

AKT/PKB: Protein kinase B
ANOVA: Analysis of variance
Ask1: Apoptosis signal-regulating kinase 1
ATF4: Activating transcription factor 4
ATF6: Activating transcription factor 6
BAK: Bcl-2 antagonist/killer
BAX: Bcl-2 associated protein X
BCAO: Bilateral common Carotid Artery Occlusion
BCL2: B cell lymphoma 2
BCL2-xL: B cell lymphoma 2 extra long
BH: Bcl-2 homology
BH3: Bcl-2 homology domain 3
Bid: BH-3 interacting domain death agonist
Caspase: Cysteine aspartic acid protease
CCA: Common Carotid Artery
CHOP: C/EBP homologous protein
DRP1: Dynamin-related protein 1
eIF2α: Eukaryotic translation initiation factor 2 alpha
ER: Endoplasmic reticulum
ERAD: ER-associated protein degradation
GADD153: Growth arrest and DNA damage-inducible 153
GAPDH: Glyceraldehayde-3-phosphade dehydrogenase
G-CSF: Granulocyte-colony stimulating factor
G-CSFR: G-CSF receptor
GRP 78: Glucose-regulated protein 78
IRE1α: Inositol-requiring protein-1alpha
JAK: The Janus kinase/Kainic acid
LC3: Light chain 3
LDF: Laser Doppler Flowmeter
MAP: Microtubule associate protein
OPA1: Optic atrophy protein 1
P-Akt: phosphorylated Akt
PERK: Protein kinase RNA (PKR)-like ER kinase
PI3K: Phosphatidylinositol-3-kinase
PUMA: p53-upregulated modulator of apoptosis
RCBF: Regional cerebral blood flow
STAT3: Signal transducer and activator of transcription
TRAF2: Tumor necrosis factor-α receptor-associated factor 2
TTC: 2, 3, 5- Triphenyl tetrazolium chloride
UPR: Unfolded protein response
XBP-1: X-Box-binding protein
