# Supplementary material for: Mode of action of granulocyte-colony stimulating factor (G-CSF) as a novel therapy for stroke in a mouse model
Source: J Biomed Sci. 2020 Jan 6;27:19. doi: 10.1186/s12929-019-0597-7 (PMC6943893; doi:10.1186/s12929-019-0597-7)

**Table S1: List of antibodies used for western blotting and immunohistochemistry.**

| **Antibody** | **Company and Catalogue Number** |
| --- | --- |
| Anti-G-CSF antibody | Abcam, Cat# ab181053 |
| Anti-GRP78 antibody | Abcam, Cat# ab21685 |
| Anti-ATF4 antibody | Abcam, Cat# ab85049 |
| Anti-Caspase-12 antibody | Abcam, Cat# ab62484 |
| Anti-OPA1 antibody | Abcam, Cat# ab157457 |
| Anti-DRP1 antibody | Abcam, Cat# ab184247 |
| Anti- PUMA antibody | Abcam, Cat# ab54288 |
| Anti-p53 Antibody | Abcam, Cat# ab16465 |
| Anti-XBP1 Antibody | Abcam, Cat# ab37152 |
| Anti-GAPDH antibody | Cell Signaling Technologies, Cat# 5174S |
| Anti-Akt antibody | Cell Signaling Technologies, Cat# 4691S |
| Anti-Phospho Akt (P-Akt) antibody | Cell Signaling Technologies, Cat# 4060S |
| Anti-Bax antibody | Cell Signaling Technologies, Cat# 2772S |
| Anti-Bak antibody | Cell Signaling Technologies, Cat# 3814S |
| Anti-Beclin-1 antibody | Cell Signaling Technologies, Cat# 3738S |
| Anti-eIF2α antibody | Cell Signaling Technologies, Cat# 2103S |
| Anti- Phospho DRP1(P-DRP1) (ser 616) antibody | Invitrogen Prod# PA5-64821 |
| Anti G-CSFR (M-20) antibody | SantaCruz Biotechnology, Cat# sc-694 |
| Anti CHOP/GADD153 antibody | SantaCruz Biotechnology, Cat# sc-793 |
| Anti-Bcl-2 (N-19) antibody | SantaCruz Biotechnology, Cat# sc-492 |
| Anti-ATF6 antibody | Imgenex, Cat#IMG-273 |
| Secondary goat anti-mouse antibody | LI-COR Bioscience, cat#925-32210 |
| Secondary goat anti-rabbit antibody | LI-COR Bioscience, cat#925-32211 |

**Figure S1: Neuroprotective effect of G-CSF Protein as determined in immunohistochemistry protein signal (a) DRP1 (b) OPA1 (c) P53.**

Analysis was conducted comparing of the protein signals seen within the middle region of the cerebrum in mice brain using the Immunohistochemistry technique. a, b, c The signal of DRP1 protein was detected to be more frequent/stronger in the vehicle animals and least in the animal treated with G-CSF after 30-minute BCAO. Sham animal is observed to be in-between. d, e, f The signal of OPA1 proteins are detected more frequent in animals treated with G-CSF in comparison to vehicle animals after 30-minute BCAO. Sham animals are seen to have no OPA1 protein signal. g, h, i P53 protein signaling is detected more frequent in vehicle animals in comparison to animals treated with G-CSF after 30-minute BCAO. Sham animals are seen to have no P53 protein signal. For all images, nuclei were counterstained with DAPI. DRP1, OPA 1 and Neu-N protein are labeled in red, P53 is labeled in green. a, d, g represents Sham; b, e, h represents BCAO and c, f, i represent BCAO + G-CSF. Scale bar = 20 μm. (n = 3)

**Figure S1**


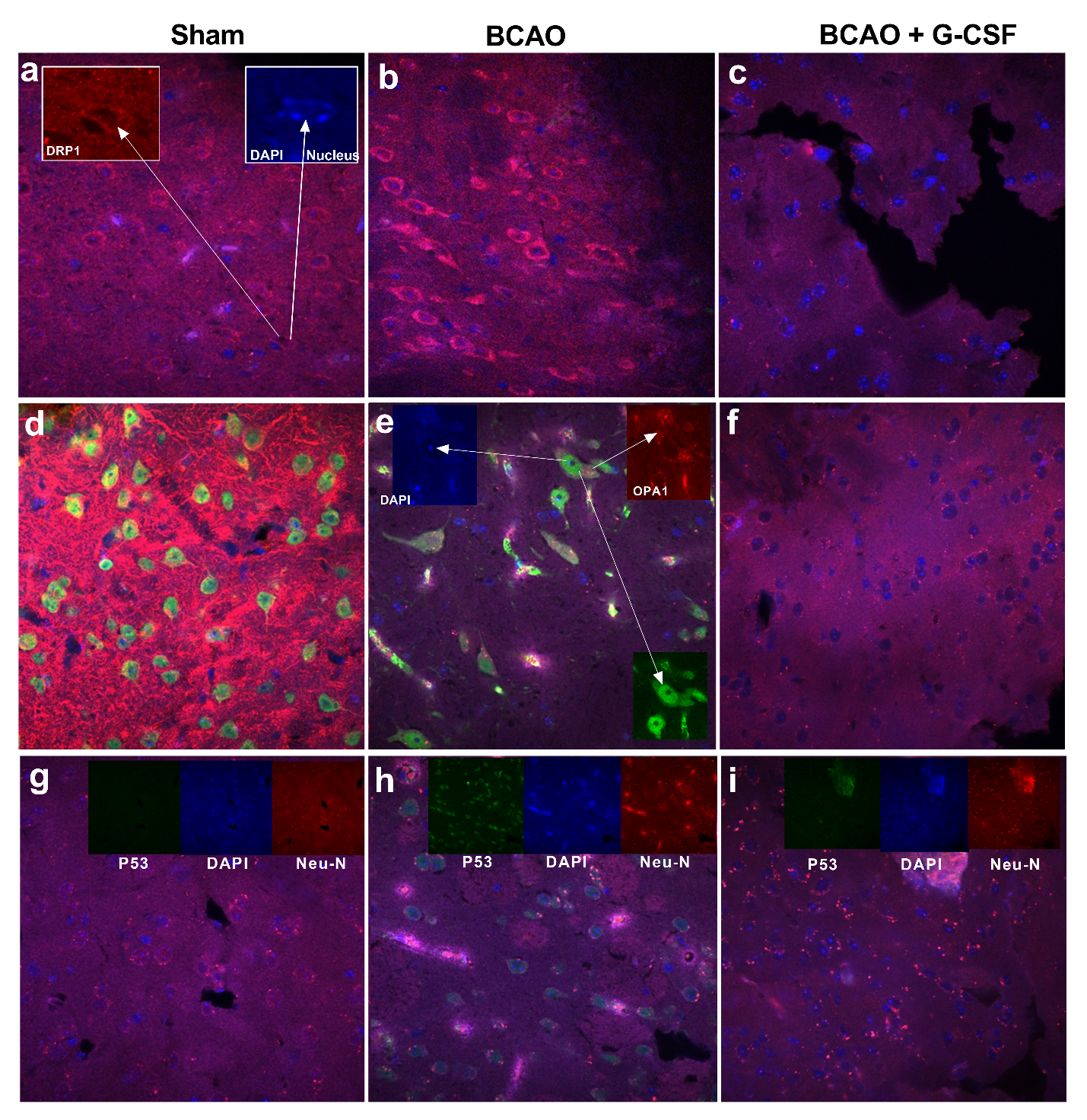

Supplement: Supplementary file 1 — Additional file 1 Table S1. List of antibodies used for western blotting and immunohistochemistry. Fig. S1. Neuroprotective effect of G-CSF Protein as determined in immunohistochemistry protein signal (a) DRP1 (b) OPA1 (c) P53. Analysis was conducted comparing of the protein signals seen within the middle region of the cerebrum in mice brain using the Immunohistochemistry technique. a, b, c The signal of DRP1 protein was detected to be more frequent/stronger in the vehicle animals and least in the animal treated with G-CSF after 30-min BCAO. Sham animal is observed to be in-between. d, e, f The signal of OPA1 proteins are detected more frequent in animals treated with G-CSF in comparison to vehicle animals after 30-min BCAO. Sham animals are seen to have no OPA1 protein signal. g, h, i P53 protein signaling is detected more frequent in vehicle animals in comparison to animals treated with G-CSF after 30-min BCAO. Sham animals are seen to have no P53 protein signal. For all images, nuclei were counterstained with DAPI. DRP1, OPA 1 and Neu-N protein are labeled in red, P53 is labeled in green. a, d, g represents Sham; b, e, h represents BCAO and c, f, i represent BCAO + G-CSF. Scale bar = 20 μm (microns). (n = 3). [file 12929_2019_597_MOESM1_ESM.docx]
